# Approaches to measure class importance in Knowledge Graphs

**DOI:** 10.1371/journal.pone.0252862

**Published:** 2021-06-10

**Authors:** Daniel Fernández-Álvarez, Johannes Frey, Jose Emilio Labra Gayo, Daniel Gayo-Avello, Sebastian Hellmann

**Affiliations:** 1 Department of Computer Science, University of Oviedo, Oviedo, Spain; 2 AKSW Group, University of Leipzig, Leipzig, Germany; University of Pisa, ITALY

## Abstract

The amount, size, complexity, and importance of Knowledge Graphs (KGs) have increased during the last decade. Many different communities have chosen to publish their datasets using Linked Data principles, which favors the integration of this information with many other sources published using the same principles and technologies. Such a scenario requires to develop techniques of Linked Data Summarization. The concept of a *class* is one of the core elements used to define the ontologies which sustain most of the existing KGs. Moreover, classes are an excellent tool to refer to an abstract idea which groups many individuals (or instances) in the context of a given KG, which is handy to use when producing summaries of its content. Rankings of class importance are a powerful summarization tool that can be used both to obtain a superficial view of the content of a given KG and to prioritize many different actions over the data (data quality checking, visualization, relevance for search engines…). In this paper, we analyze existing techniques to measure class importance and propose a novel approach called ClassRank. We compare the class usage in SPARQL logs of different KGs with the importance ranking produced by the approaches evaluated. Then, we discuss the strengths and weaknesses of the evaluated techniques. Our experimentation suggests that ClassRank outperforms state-of-the-art approaches measuring class importance.

## 1 Introduction

With the development of semantic web technologies, a huge volume of information has been published as Linked Data (LD) or Linked Open Data (LOD) in the form of Resource Description Framework (RDF) graphs. LOD is being used for a wide range of different applications, including search engines [1–3] or recommendation systems [4, 5]. Many different knowledge domains are covered by LOD datasets, and there are several projects whose main goal is to store and to offer as many general-purpose LOD content as possible. DBpedia [6], Wikidata [7], YAGO [8], and OpenCyc [9] are insightful examples.

The size and variety of such projects make their content complicated. The process of gradual discovery and understanding of the contents of a large and unfamiliar LOD source has been called *graph exploration* [10]. In such a context, automatic summarization techniques are now more necessary than ever. Providing simplified versions of a graph’s content is desirable in two ways for graph exploration. On the one hand, it allows consumers to decide whether a graph can be suitable for their purposes. On the other hand, it can be a tool to discover which are the most important topics, entities, or types of relations within a given source.

This is particularly relevant in cross-domain datasets maintained by different people, organisms, or tools. The bigger, more complex, or greater it is the number of different agents maintaining a LOD source, the harder it is to produce accurate handcrafted summaries.

There are many valid approaches to produce summaries of different natures of LOD content [11], especially for schema elements such as classes [12–14]. These summaries frequently consist of reduced graphs which are representative of the original structure. All these techniques need to identify which are the most important elements in the target graph. Importance rankings can are to produce any of the aforementioned summaries, but they can also act as a simpler summary by themselves.

The concept of *class* has a key role in these processes. Classes are abstract ideas which group many individuals sharing a common set of features under the same label. In general, RDF graphs are explored or exploited via SPARQL queries. Thus, knowing the set of properties associated to a given class allows for writing SPARQL queries involving their instances, since they all share a common structure w.r.t property usage.

Despite this, the problem of detecting class importance to elaborate class rankings has not received enough attention from the scientific community. The notion of *importance* has not been properly defined yet. In this paper, we discuss the notion of importance as opposed to the notion of *relevance* against a given purpose. Then, we evaluate different approaches to measure class importance. Our contribution is twofold:

- We make a compilation and comparison of existing unsupervised techniques to produce class importance rankings. The techniques are compared in terms of quality of their results, type of information used, and computational complexity.
- We propose a new technique called ClassRank. Our approach is based on PageRank [15] scores and assigns each class an importance score computed upon the importance of its instances. ClassRank can capture the importance of classes with few instances when those instances are important enough.

To evaluate these techniques, we applied them all over the two well-known LD sources: DBpedia and Wikidata. Then, the results produced are compared to the actual usage of each class in log samples from the official SPARQL endpoints of these two sources. Class usage in SPARQL logs has already been proposed as a reference to measure class importance [16].

In section 2, we provide and discuss some notions which are required for a good understanding of this paper’s content. In section 3, we introduce all the techniques which are used to compute class importance in our experiments. In section 4, we describe our experiments and the obtained results in a detailed way. In section 5, we discuss the results obtained during the experimentation. In section 6, we analyze some works related to ours. We focus mainly on techniques to measure class importance/relevance not used in our experiments. Finally, in section 7, we provide the conclusions of our work and future work lines.

## 2 Preliminary notions

### 2.1 Assertion Box and Terminological Box considerations

In this paper, we will assume that the reader is familiar with basic concepts of RDF such as URIs (U), literals (L), or blank nodes (B). An RDF graph *G* can be formally defined as a set of triples (s,p,o)∈(U∪B)×(U)×(U∪B∪L).

We can distinguish two different types of statements from a conceptual point of view, commonly referred to as Terminological Box (T-BOX) and Assertion Box (A-BOX) in the literature. T-BOX statements stand for abstract concepts, aka classes. They are used to describe schemata, and they are key elements to define ontologies. A statement such as (:City, :subclass_of, :Human_settlement) is a T-BOX example. On the other hand, A-Box statements contain instance information. They are more associated with Knowledge Graphs (KGs) containing information about actual individuals and how these individuals are linked between themselves and their respective schema elements. Statements such as (:New_York, rdf:type, :City) or (:New_York, :twinned_city, :Madrid) are A-BOX examples.

The techniques applied in this paper always aim to get scores and rankings for classes, not instances. However, to obtain such a result, some of them consider structures purely composed of T-BOX statements, while some others use knowledge related to the A-BOX part of the graph as well.

### 2.2 Importance vs relevance

Discovering the most important nodes in a graph has been proven as a key task in order to perform further actions, such as summarization or priorization of contents. Even with that, the notion of importance itself remains ambiguous. Several metrics that compute different topological features have been purposed to determine the importance of elements. However, there is not an approach that outperforms the rest in general terms capturing the notion of node importance. The suitability of the different available techniques may depend on the planned usage for the obtained rankings.

By contrast, the notion of relevance is linked to a purpose, therefore it is much easier to define within a context. For instance, search engines provide rankings of elements w.r.t. its relevance to a given query. Recommendation systems produce lists with the most relevant items for their users. Different classification systems may produce different groupings attending to different criteria, so the algorithms used check the relevance of each element against those criteria.

Relevance is not just easier to define, but also to evaluate. Both search engines and recommendation systems are designed to be used by some final users, who are legitimate judges to classify a set of results as relevant or not for their expectations and preferences. Then, those users can determine whether a given classification is correct. In the case of importance decoupled of a specific purpose, the idea of what are the most important elements in a dataset may be different for the owner or creator of the source and for each one of its consumers. And there is not a definitive argument proving which one of them is right.

For our experimentation, we have adopted the idea of class importance used in [16]. The authors compare several centrality metrics against an artificial gold standard. They associate each class a score in light of how frequently that class is mentioned in a SPARQL query against an endpoint exposing the graph’s content. Further details about how to identify class mentions in the logs and how to measure class importance with them will be provided in section 4. The rankings obtained from the logs will be used as a reference to evaluate the approaches mentioned in section 3.

## 3 Metrics

### 3.1 Importance metrics applied over schema structures

In this section, we will work with a formal definition of a graph *G* = (*V*, *E*), where *V* is a set of nodes or vertexes and *E* is a set of edges linking those nodes.

The techniques introduced in this section are Degree, Betweenness, Bridging Centrality, Closeness, Harmonic Centrality, and Radiality. In our experimentation, they are applied over graphs composed only by T-Box elements. In the interest of brevity, in the following sections of this paper, we will refer to them all as Only Terminological Techniques (OTT). The techniques of this section could be theoretically applied to graphs containing A-BOX statements as well. However, except Degree, they have a computational cost that does not make them suitable to be applied over KGs as big as the ones used in this paper’s experiments.

The techniques mentioned in this section are used for general network analysis and they all aim to measure node centrality. A thorough review of these approaches and similar ones is offered in [17].

#### 3.1.1 Degree

The degree is one of the simplest measures of graph centrality. The degree of a node *e* is the number of edges incident to *e*. We will denote the degree of a node *e* ∈ *V* as *D*(*e*).

#### 3.1.2 Betweenness

The Betweenness *B*(*e*) of a node *e* is the ratio of shortest paths between any pair of nodes (*u*, *v*)/*e* ∈ *V* ∧ *e* ≠ *u* ≠ *v* that pass through *e* compared to the total number of shortest paths. Let *σ*(*G*, *e*) be the function which gives the number of shortest paths in *G* passing through *e*, and *σ*(*G*) be the total number of shortest paths in *G*. Then, the Betweenness *B*(*e*) can be defined as:

$$\begin{array}{c} B\left(e\right)=\sum_{e\neq u\neq v}\frac{\sigma (G,e)}{\sigma (G)} \end{array}$$ (1)

#### 3.1.3 Bridging centrality

A bridging path is an indirect connection between two aggregate nodes in a graph, i.e., a link of two densely connected components (e.g. a domain knowledge, an organization) via a third node known as bridging node. On the top of this concept, the Bridging Centrality *BC*(*e*) of a node *e* assigns to *e* a score which aims to measure how much *e* acts as a bridge mainly for the nodes in its neighborhood in *G*. For such a goal it combines both local and global metrics of centrality. It is based on Betweenness and the Bridging Coefficient *B*_*c*_(*e*) of a node *e*, which is defined as follows:

$$\begin{array}{c} B_{c}\left(e\right)=\frac{D{\left(e\right)}^{-1}}{\sum_{i\in N(e)}D{\left(i\right)}^{-1}} \end{array}$$ (2)

where *N*(*e*) is the set of nodes in the immediate neighborhood of *e*. With this, *BC*(*e*) is defined as:

$$\begin{array}{c} BC\left(e\right)=B\left(e\right)\cdot B_{c}\left(e\right) \end{array}$$ (3)

In the contexts of KGs, *BC*(*e*) can be used to identify useful nodes linking different information topics or knowledge domains.

#### 3.1.4 Closeness and Harmonic Centrality

The Closeness *C*(*e*) of a node *e* gives a hint about how close *e* is to every other node in *G*. The Closeness score of *e* consists of the average of the length of the shortest paths from *e* to every *v*/*v* ∈ *V* ∧ *v* ≠ *e*. The Harmonic Centrality *HC*(*e*) consists of a slight modification of Closeness, computing the harmonic mean of distances instead of the average. Let *d*(*u*, *v*) be the function that gives the length of the shortest path between *u* and *v*. With this, *HC*(*e*) can be defined as follows:

$$\begin{array}{c} HC\left(e\right)=\frac{1}{\sum_{u\neq e}d\left(e,u\right)} \end{array}$$ (4)

Harmonic Centrality and Closeness produce an inverse sorting of elements, i.e., the reverse rank of Closeness would be the same rank of Harmonic Centrality. The element with the highest score in Closeness is the less central one, i.e., the one whose paths to every other node happen to be the longest ones. By contrast, the element with the highest score in Harmonic centrality is the most central one. Since the rest of the techniques employed produce scores in which the higher is the value the more important is the element, we will use Harmonic Centrality instead of Closeness.

#### 3.1.5 Radiality

Radiality, as well as Closeness or Harmonic Centrality, aims to quantify how close is a node to all the rest in a graph. Radiality is based on the concept of Diameter of a graph *Δ*(*G*), which is the maximum distance between any pair of nodes in *V*. With this, the Radiality *R*(*e*) of a node *e* can be defined as follows:

$$\begin{array}{c} R\left(e\right)=\frac{1}{\sum_{u\neq e}\left(\Delta \left(G\right)-\left(\frac{1}{d(e,u)}\right)\right)} \end{array}$$ (5)

### 3.2 Importance metrics applied over the whole graph structure

In this section, we will introduce Instance Counting (IC), PageRank, HITS, and ClassRank. They have in common that they use T-Box and A-Box knowledge of the target KG to produce a result. In the interest of brevity, we will refer to them as Also Assertion Techniques (AAT).

Since IC and ClassRank are based on class-instance relations, they both need A-BOX statements for their computation. In opposition, PageRank and HITS can be applied over any directed network structure, so they can work as AAT or OTT. In this paper, we analyze the results of applying HITS and PageRank over the T-BOX subgraph and the whole KG. We will refer to these computations as PageRank/HITS OTT when they are applied over the T-BOX subgraph, and as PageRank/HITS AAT when they are applied to the whole KG.

#### 3.2.1 Instance Counting

This importance metric is tightly linked to the RDF world and, specifically, to the class-instance relation. The more instances a class has, the more important the class is. Several public and widely-used data sources offer statistics about the number of instances as a clue of class importance, such as Wikidata [18], or offer separate files in their dumps to manage triples about instantiation, such as DBpedia [19]. Instance Counting (IC) is a simple and scalable importance metric.

Typically, in RDF sources, the relation of instance-class between two elements *e*_*c*_ and *c* is expressed using the property *rdf:type* in a triple (*e*_*c*_, rdf:type, *c*) (all the prefixes used in this paper are commonly used and can be solved using the on-line tool *prefix.cc* [20]). However, properties with a similar semantic to *rdf:type* can be used, such as *‘P31—instance of’* in Wikidata.

#### 3.2.2 PageRank

PageRank is based in a notion of importance which can be informally explained with the next statement: an element gains importance if it receives more links form other elements, if those links come from important elements, and if those elements have few outgoing links. PageRank scores are values in [0, 1] with a nice statistical interpretation. The PageRank score of a node *e* is the probability that a random surfer, starting at a random node and jumping from node to node following links, stops at node *e*. PageRank was originally designed to rank the importance of pages in the World Wide Web. Ro model the actual behavior of an Internet user that may follow links between pages, but may write as well some new URL in his browser to move to a page non-linked from the current one, PageRank uses a parameter *α*. The probability *d* that the random surfer has of getting bored of following links and jumps to a random page is *d* = 1 − *α*.

#### 3.2.3 HITS

The HITS algorithm [21] assigns two types of scores to the nodes in a graph: hub score and authority score. A given node will have a greater authority score when it receives links from nodes with a high hub score. Also, a given node will have a greater hub score when it points to nodes with a high authority score.

As well as PageRank, HITS is an algorithm designed to rank the importance of pages in web search contexts. The hub score provided by HITS is actually a similar notion to PageRank: in both cases, a node gains importance when it receives links from important nodes. However, PageRank and HITS consider a different notion of importance for those incoming links. The authority score aims for finding pages that link to many other important nodes. This notion can be very useful for web search tasks: instead of looking for the most relevant result, it finds nodes from which you can jump to many other relevant results.

HITS is usually applied over subgraphs of a certain network containing nodes and edges relevant to a given query. For example, when applied to web search, HITS does not compute the hub and authority score for each page on the Internet. Instead, it computes the connections between those pages that are relevant to a query according to some other technique. Thus, both hub and authority scores are relevant to the nature of that query, and not just to the importance w.r.t to the whole network structure.

Despite this, HITS can be applied over the whole KG, and so we do in this paper. When applying HITS, we will use the hub score in every case, which is the one whose semantic is closer to the notion node importance.

#### 3.2.4 ClassRank

We propose a novel technique to compute class importance called ClassRank. The ClassRank score of a class *c* consist of the aggregation of the PageRank scores of its instances. Let *PR*(*e*, *α*) be the PageRank of *e* with a damping factor of *α*. And let *I*(*c*) be a function which gives the set of all the instances of the class *c*. Then the ClassRank score *CR*(*c*, *α*) of a class *c* with a damping factor of *α* can be simply defined as follows:

$$\begin{array}{c} CR\left(c,\alpha \right)=\sum_{e_{c}\in I\left(c\right)}PR\left(e_{c},\alpha \right) \end{array}$$ (6)

As well as IC, ClassRank qualifies the importance of a class w.r.t. their instances. Nevertheless, while IC purely quantifies the number of instances, ClassRank is able to keep a balance between the quantity and quality (aka importance) of those instances.

ClassRank assigns scores in [0, 1] which also has a nice statistical interpretation. *CR*(*c*, *α*) is the probability that a random surfer such as the one described for PageRank has to land in an instance of *c*.

Let *V*_*C*_ be the subset of nodes in *V* which are classes. While it is always true that ∑_*e*∈*V*_ *PR*(*e*, *α*) = 1, ClassRank does not have a similar property, i.e., it is not always true that $\sum_{c\in V_{C}}CR\left(c,\alpha \right)=1$. The PageRank scores of nodes with no classes are never used, while the score of nodes with more than one class are used to increase the ClassRank score of all its classes. Then, to make $\sum_{c\in V_{C}}CR\left(c,\alpha \right)=1$ true, every node *e*/*e* ∈ *V* ∧ *PR*(*e*, *α*) > 0 should have exactly one class.

To decide which instances give their score to a given class, ClassRank relies on the concept of *class-pointer*. Usually, each KG uses a single property to express instance-class relations, being *rdf:type* the usual choice for such a goal. In those graphs, a straightforward pick of class-pointer is *rdf:type*. However, there are occasions in which the user may find it useful to use a more flexible notion of instance-class by picking different properties.

An example of such an arguable property could be *:occupation*. Let’s consider a triple (:sarah, :occupation, :doctor) representing that someone called Sarah works as a doctor. It cannot be said that the essential type of *:sarah* is *:doctor* but, even with that, it can be said that Sarah *is a* doctor in informal speech. Let *G* be a graph describing many people’s job, where the *rdf:type* of all the individuals is *:Person*. Then, choosing *rdf:type* as class-pointer to compute *G* with ClassRank will produce not really useful results. The only class with instances would be *:Person*. However, an execution of ClassRank using *:occupation* as class-pointer, or *rdf:type* and *:occupation* at a time, will give a distribution of importance among the different occupations (classes) stated.

Some other scenarios in which it can be interesting to choose class-pointers different from *rdf:type* are ontologies or KGs where most of the knowledge is T-BOX. Then, properties such as *rdfs:subClassOf* could be used to propagate the importance of subclasses to their parent classes.

To support those cases and similar ones, we define class-pointers as properties that are supposed to be used in triples where the object is a class. This definition does not imply that there must be a strict instance-class relation between a pair of elements linked by a class-pointer. However, as that is the usual case, and to avoid verbosity, in this paper, we will use the term instance when referring to elements that give its PageRank score to a class. ClassRank can use several class-pointers in a single execution to adapt to the user needs.

Even if ClassRank is inspired and built over PageRank scores, it is important to remark that *PR*(*c*, *α*) ≠ *CR*(*c*, *α*). While PageRank measures the importance of the URI of a class within a graph, ClassRank uses this URI as a pure label to represent the accumulated importance of a group of elements whose common feature is having the same class. Actually, as it is defined, *PR*(*c*, *α*) does not have any effect on *CR*(*c*, *α*) unless *c* is its own instance. Formally stated, *PR*(*c*, *α*) does not have any effect on *CR*(*c*, *α*) unless it is true that (*c*, *p*, *c*) ∈ *G* ∧ *p* ∈ *CP*(*G*), where *CP*(*G*) is the set of class-pointers of *G*.

ClassRank’s pseudo-code has been formalized in Algorithm 1. However, some of the conventions used must be described. We define a graph *G* as a set of triples *G* = {*t*_1_, *t*_2_…*t*_*n*_}. A triple *t* is a group *t* = (*s*_*t*_, *p*_*t*_, *o*_*t*_) (subject, predicate and object). We use the macro *f*_*PR*_(*G*, *α*) to refer to the standard PageRank function. *f*_*PR*_(*G*, *α*) receives a graph *G* and a dumping factor *α* as input, and it returns a vector of size *n*, being *n* the number of nodes contained in *G*. We use *E* to denote the set of nodes contained in *G*, and *P* to denote the set of properties used in any *t* ∈ *G*. We denote the set of classes to be classified with *E*_*C*_, and the set of class-pointer properties with *P*_*C*_. We use *f*_∅_ to denote an empty function *f*_∅_: ∅ → ℘(*E*_*C*_), i.e., a function whose domain is the empty set ∅ and whose co-domain consist of all possible subsets (powerset) of *E*_*C*_.

**Algorithm 1** ClassRank pseudo-code

**Input**: *G* = Target Graph

**Input**: *P*_*c*_ = Set of properties identified as class-pointer

**Input**: *α* = Damping factor

**Input**: *θ* = Security threshold

**Input**: *E*_*C*_ = Target classes (it can be an empty set)

1: *I*_*c*_ ∅;

2: $Q\leftarrow \begin{array}{ll} & \begin{array}{cccc} o_{1} & o_{1} & \cdots & o_{1} \end{array}\\ \begin{array}{c} p_{1}\\ p_{2}\\ \vdots \\ p_{n} \end{array} & \left(\begin{array}{llll} 0 & 0 & \ldots & 0\\ 0 & 0 & \ldots & 0\\ \vdots & \vdots & \ddots & \vdots \\ 0 & 0 & \ldots & 0 \end{array}\right) \end{array}$                   ▷Stage 1

3: *L* ← *f*_*PR*_(*G*, *α*)                          ▷Stage 2

4: **for each** $t_{i}=\left(s_{t_{i}},p_{t_{i}},o_{t_{i}}\right)\in G$ **do**

5:  **if** $p_{t_{i}}\in P_{c}$ **then**

6:   $Q_{p_{t_{i}},o_{t_{i}}}\leftarrow Q_{p_{t_{i}},o_{t_{i}}}+1$

7: **if** *E*_*C*_ ≠ ∅ **then**

8:  *I*_*c*_ ← *E*_*C*_

9: **else**

10:  **for each** *j* ∈ [1, |*E*|] **do**

11:   **for each** *i* ∈ [1, |*P*_*c*_|] **do**

12:    **if** $Q_{p_{i},o_{j}}>\theta$ **then**

13:     *I*_*c*_ ← *I*_*c*_ ∪ {*o*_*j*_}

14:     **break**                          ▷Stage 3

15: $L^{\prime }\leftarrow (\begin{array}{llll} \overset{e_{c_{1}}}{0} & \overset{e_{c_{2}}}{0} & \overset{\cdots }{\ldots } & \overset{e_{c_{n}}}{0} \end{array})$

16: $S\leftarrow (\begin{array}{llll} \overset{e_{c_{1}}}{f_{\emptyset }} & \overset{e_{c_{2}}}{f_{\emptyset }} & \overset{\cdots }{\ldots } & \overset{e_{c_{n}}}{f_{\emptyset }} \end{array})$

17: **for each** $t_{i}=\left(s_{t_{i}},p_{t_{i}},o_{t_{i}}\right)\in G$ **do**

18:  **if** $p_{t_{i}}\in P_{c}\wedge o_{t_{i}}\in I_{c}\wedge Q_{p_{t_{i}},o_{t_{i}}}\geq \theta$ **then**

19:   **if** $p_{t_{i}}\notin D\left(S_{o_{t_{i}}}\right)$ **then**

20:    $G\left(S_{o_{t_{i}}}\right)\left[p_{t_{i}}\right]\leftarrow \emptyset$

21:   $s_{t_{i}}\notin \overset{a\in D(S_{o_{t_{i}}})}{⋃}S_{o_{t_{i}}}\left(a\right)$

22:    $L_{o_{t_{i}}}^{\prime }\leftarrow L_{o_{t_{i}}}^{\prime }+L_{s_{t_{i}}}$

23:   $G\left(S_{o_{t_{i}}}\right)\left[p_{t_{i}}\right]\leftarrow G\left(S_{o_{t_{i}}}\right)\left[p_{t_{i}}\right]\cup \left\{s_{t_{i}}\right\}$

**Output**: *L* = PageRank score of each entity

**Output**: *S* = instantiation vector

**Output**: *L*′ = Aggregated PageRank score of each class

In line 16, we initialize a vector of maps, and to represent each map we are using function notation. Given a certain function *f*, we denote its domain with D(f), and its graph with G(f). We modify the definition of a function *f* by adding or modifying elements in G(f), i.e., in order to define *f*(*a*) = *b*, we will use G(f)[a]←b.

Algorithm 1 receives as input a target graph *G*, a set *P*_*C*_ of class-pointers, a damping factor *α* used for the PageRank execution, a security threshold *θ*, and a set of target classes *E*_*C*_, which can be empty if the target classes are not known a priori.

The threshold *θ* is used to ignore classes with few instances. This is specially useful when the classes to rank are not known a priori and the user of ClassRank wants to discover those classes using the classpointers, but he prefers to discard those elements with few instances.

The algorithm returns three results: 1) The standard PageRank vector for every entity {*e*/*e* ∈ *E*}, denoted as *L*; 2) the ClassRank vector for every class {*e*_*C*_/*e*_*C*_ ∈ *E*_*C*_}, denoted as *L*′, and 3) a matrix containing information about which entities point to which classes using which class-pointer, denoted as *S*. *L*′ provides the importance of each class, while *S* allows to analyze the source of that importance.

We have divided ClassRank in three stages preceded by a preliminary one to prepare some data structures.

*Preliminary stage: Initializations*. At this stage, we initialize some data structures that will be used during the calculations of the ClassRank scores.

*I*_*c*_ is a set that will contain identifiers of the ranked classes. *Q* is a matrix of (*m* ⋅ *n*), where *m* = |*E*| and *n* = |*P*_*C*_|. In *Q*, we annotate how many times a given object *o*_*j*_ is linked with a given class-pointer *p*_*i*_.

*Stage 1: PageRank*. At this stage, we calculate the internal relevance of each entity in *G* and we store it in vector *L*. The computation of PageRank is a widely studied problem [22, 23]. Also, there are standard libraries for many widely-used programming languages to compute it. Our implementation of ClassRank is written in Python and the PageRank computation is based on the networkx library [24]. Nevertheless, to cope with huge graphs, we have implemented a memory-optimized version of networkx’s PageRank just for non-weighted computations [25]. This implementation could be needed to reproduce the experiments proposed in this paper.

*Stage 2: Class-pointer matrix and class detection*. This stage could be divided into two phases.

In lines 4-6, the matrix Q is filled with values. This matrix contains the number of times that each classpointer is used to link each node of the graph.

In lines 9-14, we perform class discovery if needed. When *E*_*C*_ ≠ ∅, it means that the set of classes to be ranked is known a priori, so there is no need to execute lines from 10-14. Otherwise, the algorithm looks for nodes that are pointed at least *θ* times by at least a class-pointer. The set *I*_*c*_ is filled with the nodes fitting that condition. Those nodes are considered classes by the algorithm.

The security threshold *θ* has been introduced to filter wrong identifications of classes causing noise. This is especially handy in sources maintained by many agents making small editions, where human actions can cause marginal mistakes. This threshold should be used carefully, since it may also cause a certain number of false negatives for all those actual classes that are pointed less than *θ* times by a class-pointer.

*Stage 3: ClassRank scores*. The ClassRank score of each class is calculated as the aggregation of the PageRank scores of its instances in lines 15 to 23. This stage can be informally summarized with the next statement: if there is a high enough number of triples that have the same class-pointer as predicate and the same class URI as object, then the PageRank scores of the subjects of those triples are added to the ClassRank score of the class URI.

In lines 17-18, for each triple $t_{i}=\left(s_{t_{i}},p_{t_{i}},o_{t_{i}}\right)$, we check whether $o_{t_{i}}$ is a class and $p_{t_{i}}$ is a class-pointer linked to $o_{t_{i}}$ at least *θ* times. If this is true, we perform three actions:

- In lines 19-20 we include $p_{t_{i}}$ as class-pointer of $o_{t_{i}}$ in the vector of maps *S*, just in case it had not been already included.
- In lines 21-22 we add the PageRank score of $s_{t_{i}}$ to the ClassRank score of $o_{t_{i}}$, just in case it had not been already added.
- In line 23 we specify in the vector of maps *S* that $s_{t_{i}}$ is instance of $o_{t_{i}}$ due to the class-pointer $p_{t_{i}}$.

There is a public implementation of ClassRank available in a GitHub repository [26].

### 3.3 Adapted importance metrics

The authors in [16] propose an approach to adapt the OTT metrics described in section 3.1. This adaptation incorporates A-BOX knowledge to compute the scores, so the approaches become AAT. Let *T*_*i*_ be a given importance metric. In order to compute an adaptation $T_{i}^{\prime }$ of *T*_*i*_, the authors first propose a score normalization *N*(*T*_*i*_(*e*)) for a given node *e* in a scale [0, 1], defined as follows:

$$\begin{array}{c} N\left(T_{i}\left(e\right)\right)=\frac{T_{i}\left(e\right)-min\left(T_{i},G\right)}{max\left(T_{i},G\right)-min\left(T_{i},G\right)} \end{array}$$ (7)

Where *min*(*T*_*i*_, *G*) is the value of the less important node in *G* according to *T*_*i*_, and *max*(*T*_*i*_, *G*) is the value of the most important node.

The adapted metric $T_{i}^{\prime }$ is computed as follows:

$$\begin{array}{c} T_{i}^{\prime }\left(e\right)=N\left(T_{i}\left(e\right)\right)+N\left(IC\left(e\right)\right) \end{array}$$ (8)

Where *IC*(*e*) is the number of instances of class *e*. This adapted metric is an equally weighted addition of the normalized scores of IC and *T*_*i*_.

We have adapted all the OTT techniques mentioned in section 3.1 according to this formula. Besides, we have experimented with adapted versions of the rest of the techniques analyzed which are compatible with this proposal. We have combined the IC scores with PageRank OTT/AAT, HITS OTT/AAT, and ClassRank.

## 4 Experiments

We have produced reference rankings based on class usage in SPARQL logs of DBpedia and Wikidata to evaluate the mentioned metrics. Firstly, we describe the general methodology to build those reference rankings and to perform an evaluation. After that, we describe in detail each source, their associated information, and methodology adaptations for each one when needed.

### 4.1 Methodology

As suggested in [16], we have considered mentions class mentions in SPARQL queries as a reliable metric of how important a class is. We annotate a mention class in a query when:

- The URI of the class is mentioned.
- The URI of an instance of the class is mentioned.
- The URI of an element *e* is mentioned, in case *e* is used in a triple with a property whose domain/range forces *e* to be an instance of a class. Let *p*_*I*_ be a property which links an instance to its class; let *d*(*p*) and *r*(*p*) be the domain and the range of the property *p*; and let *G* be the KG under analysis. Then, formally, a class *c* is considered to be mentioned in a query if *e* is mentioned and it is true that ((*e*, *p*_*a*_, *o*) ∈ *G* ∧ *c* ∈ *d*(*p*_*a*_)) ∨ ((*s*, *p*_*b*_, *e*) ∈ *G* ∧ *c* ∈ *r*(*p*_*b*_)), even if (*e*, *p*_*i*_, *c*) ∉ *G*.

With these criteria, we elaborate class rankings based on the number of class mentions. Then, we use those lists as a gold standard to compare with the rankings produced by the techniques under evaluation.

#### 4.1.1 Reference rankings: Human-generated traffic vs machine-generated traffic

We produce two different lists for each studied source. One considers every entry available in the logs. The other one computes just those entries associated with human-performed queries. With requests made by humans we mean requests caused by people writing and executing ad-hoc SPARQL queries, or performing small tasks in some applications that trigger a single/few queries. Usually, machine traffic generates much more requests than human-performed traffic. To provide some revealing numbers, the sum of requests performed by the top-10 most active IPs in our DBpedia’s log, which is associated with machine agents, represents 44% of the total requests. If we consider the top-100 IPs, the number grows to 77% of the total.

Human traffic is not a more reliable notion of importance than machine traffic, nor vice-versa. However, it is relevant to check the difference between human-generated traffic and machine one due to the notorious impact that very few machine agents usually have over the final results. The notion of importance purely based on human actions seems to adopt a more general point of view, not so polarized by automatic voracious consumers of the endpoint. Distinguishing between requests performed by humans and requests performed by bots or applications has already been purposed in some other studies of SPARQL logs [27].

To avoid verbosity, in this paper, we will use the following abbreviations:

- Human Hosts (HH) to denote log entries associated with humans.
- Machines Hosts (MH) to denote log entries associated with machines.
- Every Host (EH) to denote all log entries.

#### 4.1.2 How to compare the rankings: Rank-biased overlap

The compared rankings have two peculiarities. First, it is feasible to have tied elements. Second, the significance of changes in the top of the ranking is higher than changes in the low spots. Search engine results or classification in sports are insightful examples of these kinds of lists. When comparing two search engines, the first results shown to the user are much more relevant than the ones in position 100^th^. Similarly, the event of a player climbing from the second seed to the top seed in a given sport receives more social attention than any other jump in deeper regions of the ranking. Importance rankings in RDF sources are used to prioritize some elements for different tasks or to get a general idea about the content of a given source. Then, the top-ranked elements are more relevant than the low-ranked ones.

It is desirable to use a metric that can compare the similarity of two rankings naturally handling these two features. We have found that Rank-Biased Overlap [28] fits our requirements. Originally, RBO is defined as a distance measure between two rankings, where 0 means minimum distance and 1 means maximum distance. However, it can be trivially transformed into a metric by calculating 1 − *RBO*, where 1 means maximum similarity, and vice-versa. From this point, we will work with the definition of RBO as a metric.

Essentially, RBO checks the overlap of two rankings at incrementally increasing depths. The elements checked at each depth *d* are those in rank [1,2…d]. Since the first element will be checked looking for overlap at every iteration, this element has the greatest impact on the final results. The following element with more importance over the score will be the second one, and so on. At each iteration, RBO computes the ratio of overlapped elements. It produces a result by adding all those ratios weighted using an infinite series of weights whose sum converges always to a fixed value. The weights can be configured to give a certain amount of importance to a region of the top rank using a parameter *p*.

The *p* parameter has a nice statistical interpretation. It models the user’s persistence when performing a manual checking of the rankings. Low values of *p* arbitrarily decrease the probability that a user has to keep exploring ranks, and vice-versa. The extreme case *p* = 0 causes that the only position checked is the first one. With *p* = 0, RBO gives a result of 0 (no overlap) when the first element of both rankings is not the same, or 1 (perfect overlap) on the contrary case. The rest of the ranking would be ignored. The higher is the value of *p*, the less probable it is that the user stops exploring the ranking.

Greater values of *p* arbitrarily increase the importance of wider prefixes of the rankings. Each iteration *k* will always have a greater impact over the results than *k*+ 1, but greater values of *p* decrease that difference. *p* can also be interpreted as a parameter to configure the exact amount of importance over the final score that a given prefix length has. For instance, a value of *p* ≃ 0.9 gives an importance of 86% to the top 10 elements. This means that the sum of weights of the first 10 iterations of RBO will be 0.86. Although there is not a function to obtain a value of *p* for a couple of chosen values of importance and prefix length, the authors in [28] provide the following useful equation:

$$\begin{array}{c} W_{RBO}\left(1:d\right)=1-p^{d-1}+\frac{1-p}{p}\cdot d\cdot (\ln\frac{1}{1-p}-\sum_{i=1}^{d-1}\frac{p^{1}}{i}) \end{array}$$ (9)

In Eq 9, *d* is the depth or prefix length, and *W*_*RBO*_(1 : *d*) is the accumulated weight of a ranking in positions 1 to *d*.

We have developed a script *f*_*p*_(*d*, *w*, *θ*_*e*_) which receives a length *d*, a weight *w*, and an error threshold *θ*_*e*_, and it returns a value *p* which approximately solves the Eq 9 for *d* and *w* = *W*_*RBO*_(1 : *d*). The script computes Eq 9 for *d* with different values of *p*, obtaining each time a result $w_{p_{i}}$. The script stops when it founds a $p_{i}/\left|w_{p_{i}}-w\right|<\theta_{e}$, and returns *p*_*i*_. This script allows us to find accurate enough values of *p* for any chosen pair of prefix length and accumulated weight.

The sum of the weights at each depth of RBO always converges to a fixed value. This makes RBO an adequate candidate to compare infinite rankings without having the infinite tail’s importance dominating the finite head. When computing RBO for a given depth, even if this depth is the size of the compared rankings, the algorithm produces two results: *rbo*_*min*_ and *rbo*_*res*_. The value *rbo*_*min*_ is the overlapped score obtained after having checked the target rankings until depth *d*. *rbo*_*res*_ is the residual score that would have been added to the result in case the explored rankings had infinite but equal and equally sorted elements beyond depth *d*. With this, we can have that the max possible score for infinite lists is *rbo*_*max*_ = *rbo*_*min*_ + *rbo*_*res*_.

Then, RBO can be defined as a function *f*_*RBO*_(*R*, *L*, *p*, *d*)→*rbo*_*min*_, *rbo*_*res*_. It compares two rankings *R* and *L* until depth *d*, with a user persistence modeled by *p*, and returns a score *rbo*_*min*_ in the range [0, 1], and a residual *rbo*_*res*_ based on the assumption that *R* and *L* can have infinite elements.

The authors in [28] provide a formula to express RBO as a single point *rbo*_*ext*_ instead of a range. This formula extrapolates the tendency observed until depth *d* and assumes that it will stay stable along the infinite tail and provide an score *rbo*_*ext*_ where *rbo*_*min*_ ≤ *rbo*_*ext*_ ≤ *rbo*_*max*_. In our experimentation, we will use *rbo*_*ext*_ to obtain a single score point of similarity between two rankings.

The rankings compared will always have the same number of elements, but the way in which ties are handled may cause that they do not have the same number of ranks. When two elements have an identical score within the same ranking, they are both assigned to rank *k*, and the element after them is placed at rank *k* + 1. This means that the total number of ranks could be smaller than the total number of elements. In our experimentation, we will always execute RBO with the longest possible depth, i.e., the depth of the ranking with more ranks.

A deeper discussion about the convenience of this technique in scenarios similar to ours, as opposed to classic approaches such as Spearman [29], in which all the elements have the same impact over the final score, is provided in [28].

### 4.2 Describing sources

#### 4.2.1 DBpedia

We have used the English chapter of DBpedia in our experimentation. The contents offered in DBpedia’s SPARQL can be downloaded as text files. These logs are described in a GitHub repository and can be downloaded and used with a CC-BY license [30]. The files used contain the version of the English DBpedia chapter at the moment in which the logs were generated.

The classes ranked are the ones in the DBpedia ontology [31]. Also, the graph used to compute the OTT techniques has been the DBpedia ontology itself.

*Logs*. We have been able to mine logs of the DBpedia SPARQL endpoint of 14 different random days during 2017. The log entries are split into fourteen files. Each file includes every SPARQL request against the endpoint within a single day. The main features of the log files are provided in Table 1.

**Table 1:** Statistics about the DBpedia SPARQL logs used.

Log size	58.771GB
N° of entries in the log	74,281,130
N° of MH entries	74,187,809
N° of HH entries	93,321
N° of total class mentions	80,562,206
N° of direct class mentions	42,788,969
N° of instance mentions	33,609,979
N° of class mentions inferred by domain/range	4,163,258

We have computed class mentions using the criteria described at the beginning of this section. The scripts used to perform such a mining task are publicly available in a GitHub repository [32].

Each line in the logs contains data related to a single request to the endpoint. The version of the logs that we were able to compute was filtered and anonymized to preserve users’ privacy. We could use the following information for each entry:

- Hashed IP from where the request was performed.
- HTTP request. SPARQL queries are embedded in GET requests.
- Timestamp of the request truncated to hour precision.
- HTTP status code (200 OK, 404 Not found, 5XX server error…).

When mining logs, there is not a perfect technique to distinguish between human and machine search sessions, or even to properly perform the task of identifying a single search session. Also, the most accurate approaches use to rely on information that is not available in the version of the logs that we have been able to analyze, such as user agent, more precise timestamps, or user identifiers. For instance, authors in [27] distinguish between queries performed by humans, which they call *organic*, and queries performed by automatics processes, which they call *robotic* queries. The SPARQL logs that they use do not contain IPs, but they do include user-agents. They use this field to detect browser user-agents, which are usually connected to organic queries, and some other agents linked to known applications used by humans.

In our scenario, we combined the information of hashed IP and timestamp to detect hosts that seem to have a human-like amount and rate of requests. We picked an arbitrarily low amount of requests by hour within a single day. Any IP showing a request rate under that threshold was classified as belonging to organic agents. The chosen threshold has been 2.

We are aware that there are several situations in which this heuristic and this arbitrary threshold may cause false positives and false negatives, such as the following ones:

- A true human agent produces too many requests within a single day, which discards not just the requests of that day but also every log entry related to the same IP.
- A machine agent produces a low enough number of requests every day.
- An IP is linked to different routers on different days due to, for instance, Dynamic Host Configuration Protocol (DHCP) changes.

The mentioned issues are hard to prevent without more precise information about each log entry. However, the threshold has been chosen to pick IPs with high chances of belonging to human agents by sacrificing recall but yet having a representative sample of human-related entries.

*OTT metrics*. In this section, we describe how we computed techniques over a subgraph containing just T-BOX statements. This subgraph is the version of the DBpedia ontology corresponding to the time in which the logs were generated.

The authors in [16] use Degree, Betweenness, Bridging Centrality, Harmonic Centrality, and Radiality to measure class importance by applying them to graphs containing only T-BOX statements. In our paper, we used the same approach for those techniques. Except for Degree, whose complexity is linear to the number of nodes, all the aforementioned techniques need the computation of the shortest paths between all the nodes in the graph. This requires at least a computation time of *O*(*V* ⋅ (*V* + *E*)) [16], being *V* the number of nodes and *E* the number of edges. Thus, these algorithms are hard to compute in a source such as the analyzed section of the English chapter of DBpedia, with more than 110M triples.

Also, we have applied HITS and PageRank over the DBpedia ontology. The damping factor for the PageRank execution was set to *α* = 0.85, which is the most usual configuration of PageRank [33].

*AAT metrics*. We have applied HITS, PageRank, IC, and ClassRank over the whole KG.

Even if Degree’s complexity is low enough to execute this technique as AAT, we have not included its computation in this paper because of its similitude with IC. While IC only considers links class-instance, Degree computes every incoming or outgoing link to rank a class. However, for the top positions of that ranking, the vast majority of those links are the instance-class relations accounted by IC, which leads to nearly identical results of these two techniques.

To build the ranking of classes of PageRank, we filtered all the A-BOX elements in the obtained PageRank vector and sorted the remaining T-BOX terms in decreasing order w.r.t. to its score. The damping factor for the PageRank execution was set to the standard value *α* = 0.85.

The ClassRank scores are built on top of the PageRank ones described in the previous paragraph, so the setting *α* = 0.85 was used. When executing ClassRank, the set of target classes is known a priori. As a consequence, there was no need to perform class discovery in stage 2 of Algorithm 1. The property *rdf:type* was the only class-pointer considered. Since the only property linking an A-BOX term with any element in the DBpedia ontology is *rdf:type*, this is a straightforward decision in the context of our experiment. The same decision was taken to compute IC, i.e., the only property that we considered to link an instance to its class is *rdf:type*. Also, since the set of classes to classify is known a priori, the value that makes sense for ClassRank’s security threshold is *θ* = 0. We do not want to discard any class of the DBpedia ontology from the results.

In section 3.3, we described an adaptation of OTT approaches with IC scores. We have applied this adaptation to all the techniques mentioned in section 4.2.1, but we have also computed an adaptation of PageRank, HITS, and ClassRank over the KG to combine their scores with IC scores.

*Reference rankings*. In Table 2, we include the top-20 elements of each reference list. The whole lists are publicly available [34].

**Table 2:** Top20 elements for HH and EH entries in DBpedia.

	Human Hosts		Every Host	
Pos.	Class	Mentions	Class	Mentions
**1**	dbo:Album	1,342	dbo:Place	16,567,840
**2**	dbo:Company	667	dbo:Airport	16,082,487
**3**	dbo:Place	603	dbo:CareerStation	4,264,500
**4**	dbo:Airport	568	dbo:Band	4,065,636
**5**	dbo:Person	551	dbo:Person	3,228,266
**6**	dbo:Country	354	dbo:MusicalArtist	2,703,628
**7**	dbo:SoccerPlayer	319	dbo:Organisation	2,240,076
**8**	dbo:Settlement	301	dbo:PopulatedPlace	1,958,866
**9**	dbo:City	225	dbo:Company	1,934,215
**10**	dbo:RadioStation	178	dbo:Language	1,851,015
**11**	dbo:Film	166	dbo:Artwork	1,780,684
**12**	dbo:Writer	146	dbo:Device	1,774,061
**13**	dbo:OfficeHolder	145	dbo:Settlement	1,442,655
**14**	dbo:MilitaryConflict	129	dbo:VideoGame	1,126,766
**15**	dbo:Software	117	dbo:Album	1,014,906
**16**	dbo:MusicalArtist	114	dbo:Film	957,870
**17**	dbo:IceHockeyPlayer	113	dbo:OfficeHolder	841,060
**18**	dbo:VideoGame	108	dbo:City	834,053
**19**	dbo:Drug	108	dbo:SpTeamMember	828,302
**20**	dbo:Scientist	91	dbo:Writer	774,113

#### 4.2.2 Wikidata

Wikidata, as well as DBpedia, is a well-known general-purpose LD source. However, these two projects have some crucial differences that affect our experimentation.

Wikidata models two types of elements within its KG: entities and properties. Entities are represented with an ID starting by *Q* and followed by an integer (ex: Q5 stands for *human*; Q6256 stands for *country*). Properties are identified with a ‘P’ and an integer (ex: *P31* stands for *instance of*; *P279* stands for *subclass of*). Within the entities, we can conceptually make a distinction between classes (such as *‘Q6256—country’*) and instances (such as *‘Q30—United States of America’*).

However, there is no distinction in the way these two elements are managed. Both classes and instances are maintained by community editions and Wikidata does not provide a list of classes. It categorizes as a class any element in the KG that is an object in a triple with the property *‘P31—instance of’* or a subject/object in a triple with *‘P279—subclass of’* [35].

With this criterion, we have found 2,477,094 classes. The subgraph of elements linking those elements contains 13,791,207 triples. In opposition to the DBpedia’s case, this T-BOX subgraph is too big to apply some of the techniques mentioned in section 3.1. Specifically, Betweenness, Radiality, Harmonic Centrality, and Bridging Centrality would require huge computational power and execution time.

Wikidata barely uses any property of any external ontology. All the needed properties are defined within its own ontology and referenced with a ‘P’ ID. The class-instance relation is not expressed with *rdf:type*, but with the equivalent property *‘P31—instance of’*.

*Logs*. Wikidata made public some SPARQL logs with random samplings of valid queries in different time frames [36]. All these logs have been anonymized to preserve users’ privacy. Each log entry contains the original SPARQL query with variable names and most of the literals substituted by generic placeholders. There is also some metadata associated with the query. Wikidata classifies each entry as either ‘robotic’ or ‘organic’. A query is labeled as robotic when its user agent is not a web browser or when there is a non-human rate of queries coming from the same IP in a time span. Otherwise, the query is considered organic.

This label let us build the HH and EH rankings without further computations of the rest of the metadata. We used organic queries to elaborate the HH ranking and both organic and robotic queries for the EH ranking.

The logs used in this experimentation are the most recent ones at the moment of this writing [37]. They contain a random sample of queries performed between 2018-02-26 and 2018-03-25. A summary of the log’s content is offered in Table 3.

**Table 3:** Statistics about the Wikidata SPARQL logs used.

Log size	50,358 GB
N° of entries in the log	24,417,813
N° of MH entries	23,545,258
N° of HH entries	872,555
N° of total class mentions	105,939,034
N° of direct class mentions	22,094,776
N° of instance mentions	83,844,258

Properties in Wikidata are a more closed vocabulary compared to classes. Even if any Wikidata user can propose a new property, this proposal needs to be discussed and accepted by the Wikidata community. At the moment of this writing, a total of 8882 properties have been defined in Wikidata. Though, some of those properties are not used any longer or have been removed.

Not all the properties include domain and range restrictions in their definition. Also, some of these restrictions aim to be pure information for Wikidata users instead of actual ontological restrictions that may invalidate some triples when used wrong. Properties usually define domain and range restrictions as choice lists. For instance, *‘P106—occupation’* is defined to be used in triples whose subject should be an instance of one class in a list of 8 elements, which includes *‘Q5—human’*, *‘Q729—animal’*, or *‘Q21070598—narrative entity’*.

These domain and range definitions in some cases, and the absence of constraints in some others, do not make it possible to count class mentions in the logs via domain or range inferences. Then, when mining the Wikidata logs, we only count as class mentions 1) mentions to the class URI itself, and 2) mentions of its instances’ URIs.

*OTT metrics*. The high number of classes on Wikidata and the size of its T-BOX subgraph discard the computation of too complex techniques such as Betweenness, Radiality, Harmonic Centrality, and Bridging Centrality.

The set of techniques applied over the T-BOX subgraph of Wikidata consist of Degree, HITS, and PageRank. PageRank damping factor was set to *α* = 0.85.

*AAT metrics*. We have applied HITS, PageRank, IC, and ClassRank over the whole KG. Also, we have produced computed the adaptation with IC scores of HITS, PageRank, and ClassRank. As usual, the PageRank damping factor was set to *α* = 0.85.

We have computed several settings of ClassRank’s class-pointers *P*_*C*_ against Wikidata. Since *‘P31—instance of’* is equivalent to *rdf:type*, the most straight-forward setting is using *P*_*C*_ = {*‘P31—instance of’*}, so classes are scored w.r.t. pure instance-class relations. However, in opposition to the DBpedia case, there are many different properties linking instances and classes in Wikidata, which let use different *P*_*C*_ settings.

A class-pointer could be any property defined to be used in triples where the object is a class. However, properties in Wikidata are not defined strongly enough to find class-pointer candidates by checking their constraint definitions. Also, some properties that could be automatically detected as class-pointer candidates are not used as so in the KG. For example, *‘P413—position played on team’*, which is supposed to point to classes standing for special roles in different sports, is just used to point to classes 71.22% of the times.

Then, to obtain a list of class-pointer candidates, we computed the actual usage of every property *p* in the whole KG to obtain a ratio $r_{p}=\frac{U_{c_{p}}}{U_{p}}$, where *U*_*p*_ is the number of times that *p* is used in any triple, and $U_{c_{p}}$ the number of times in which *p* points to a class. Then, we sort the properties in descending order w.r.t. *r*. The list of properties and its associated ratio is available on-line [38]. We tested every combination of properties above a certain ratio, starting at 1.0 (the property always point to classes) and finishing at 0.5 (the property point to classes half of the times), with decrements of 0.01 for each test.

In this paper, we do not include the results of all these configurations, but just the one that we found optimal, i.e., that aligns better with the reference rankings. The best ratio for Wikidata has been *r* = 0.99. The resulting list of class-pointers with *r* = 0.99 is shown in Table 4.

**Table 4:** Properties with a class ratio ≥0.99.

P31- instance of	P8006- footedness	P4680- constraint scope
P279- subclass of	P4882- segmental innervation	P1354- shown with features
P1750- name day	P6884- target muscle	P548- version type
P1622- driving side	P1913- gene dupl. assoc. with	P4390- mapping rel. type
P8030- size designation	P4954- may prevent	P1914- gene ins. assoc. with
P3358- positive prog. pred.	P922- magnetic ordering	P2894- day of week
P8225- is metaclass for	P2352- applies to taxon	P2597- Gram staining
P6437- day of regular release	P660- EC enzyme classif.	P21- sex or gender
P5102- nature of statement	P1480- sourcing circumstances	P6216- copyright status
P6106- uses capitalization for	P2443- stage reached	P556- crystal system
P873- phase point	P1310- statement disputed by	P4794- season starts
P2308- class	P4649- id. of subj. in context	P6224- level of description
P1910- decreased expression in	P1642- acquisition transaction	P91- sexual orientation
P3357- negative diag. pred.	P4850- perm. food additive	P8127- tournament format
P2577- admissible rule in	P1917- posttranslat. mod.	P4224- category contains
P6118- season ends	P8431- course	P3150- birthday
P8115- eligible recipient	P1915- gene inv. assoc. with	P404- game mode
P970- neurological function	P1918- altered reg. leads to	P105- taxon rank
P3294- encoding	P5439- research measurement	P7937- form of creat. work

In this experimentation, we seek to rank every Wikidata element that fits in Wikidata’s definition of what a class is. Then, since the set of classes is known a priori and provided to the algorithm, the configuration of the security threshold should be *θ* = 0.

*Reference rankings*. The most important classes in Wikidata according to class usage with EH and HH entries are shown in Table 5. When comparing these rankings with the rankings produced by each technique we have discarded some special nodes from Wikidata, which are important from a structural point of view due to Wikimedia’s organizational model, but rarely used by end-users in SPARQL queries. The discarded nodes include elements such as *‘Q56005592—Wikimedia help page’*, *‘Q35252665—Wikimedia namespace’* and some other Wikimedia organizational elements.

**Table 5:** Top20 elements for HH and EH entries in Wikidata.

	Human Hosts		Every Host	
Pos.	Class	Mentions	Class	Mentions
**1**	Q5- human	312,245	Q5- human	26,902,123
**2**	Q55983715- organisms […]	261,714	Q55983715- organisms […]	3,296,393
**3**	Q515- city	215,273	Q6256- country	1,651,146
**4**	Q644371- international airport	209,245	Q3624078- sovereign state	1,553,641
**5**	Q6256- country	32,584	Q13442814- scholarly article	1,341,577
**6**	Q3624078- sovereign state	30,257	Q16521- taxon	1,306,359
**7**	Q28640- profession	21,421	Q11424- film	1,301,887
**8**	Q7270- republic	13,213	Q3947- house	1,279,807
**9**	Q12737077- occupation	11,975	Q48264- gender identity	1,231,213
**10**	Q20181813- colonial power	9,960	Q4369513- sex of humans	1,228,445
**11**	Q63791824- Baltic Sea countries	9,111	Q427626- taxonomic rank	1,013,172
**12**	Q43702- federal state	8,813	Q340169- communication medium	955,023
**13**	Q11173- chemical compound	8,519	Q3100180- rank	908,404
**14**	Q619610- social state	8,297	Q13578154- rank	907,632
**15**	Q4209223- Rechtsstaat	8,221	Q3331189- version, ed., or translat.	826,798
**16**	Q15079663- r. t. railway line	8,072	Q10876391- Wikipedia lang. edition	791,432
**17**	Q45- Portugal	7,548	Q515- city	773,382
**18**	Q11900058- explorer	7,493	Q7432- species	741,928
**19**	Q13442814- scholarly article	6,423	Q16970- church building	702,134
**20**	Q48264- gender identity	6,214	Q253019- Ortsteil	699,386

### 4.3 Results

To evaluate the similarity of two rankings *R* and *L* using different weights for their top positions, we tested several configurations of *p* in *RBO*(*R*, *L*, *p*). We used Eq 9 to obtain *p* values for different prefix sizes with a fixed importance of 0.9. In every case, the margin error to calculate *p* with the script described in section 4.1.2 was set to *α* = 0.00001.

Note that each *p* configuration solves Eq 9 for several pairs of *W*_*RBO*_(1 : *d*) and *d*. For instance, *p* ≃ 0.876343 is an adequate value for *d* = 10 and *W*_*RBO*_(1 : *d*) = 0.9, but it is also valid for *d* = 6 and *W*_*RBO*_(1 : *d*) = 0.8. We fixed *W*_*RBO*_(1 : *d*) = 0.9 just to provide understandable values of *p* instead of testing arbitrary increments.

In this paper, we have run RBO comparing the reference rankings against the rest of the metrics with several *d* values for *W*_*RBO*_(1 : *d*) = 0.9. Starting at *d* = 20, we have performed comparisons incrementing *d* in 20 spots each time until the arbitrary depth of 500, whenever this was possible. However, the relatively low number of positions in DBpedia’s HH ranking requires to choose a smaller maximum depth to explore. When building the reference rankings, some elements receive the same number of mentions. These ties, as explained in section 4.1.2, can cause that the number of ranks in a ranking can be lower than the number of total elements.

The total number of classes to rank in the DBpedia ontology is 827. With HH entries, there are many ties due to classes with few mentions or no mention at all in the logs (there are unmentioned 583 elements). This situation produces a ranking with just 62 spots.

For this reason, just for the case of DBpedia’s HH entries, we start the evaluation at the minimum prefix length of 10 and made increments of 5 spots until the arbitrary depth of 60.

**Fig 1:**
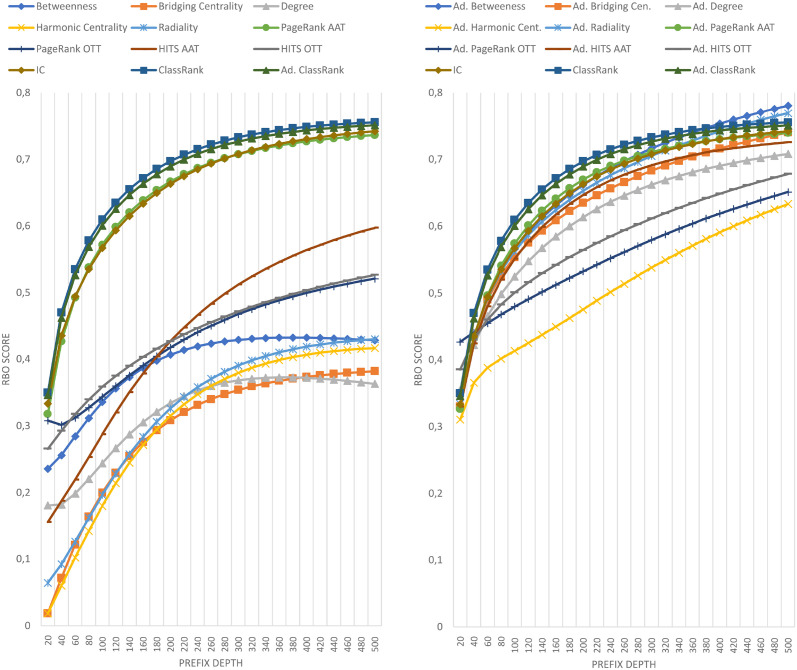
Comparison of techniques against EH log in DBpedia.

**Fig 2:**
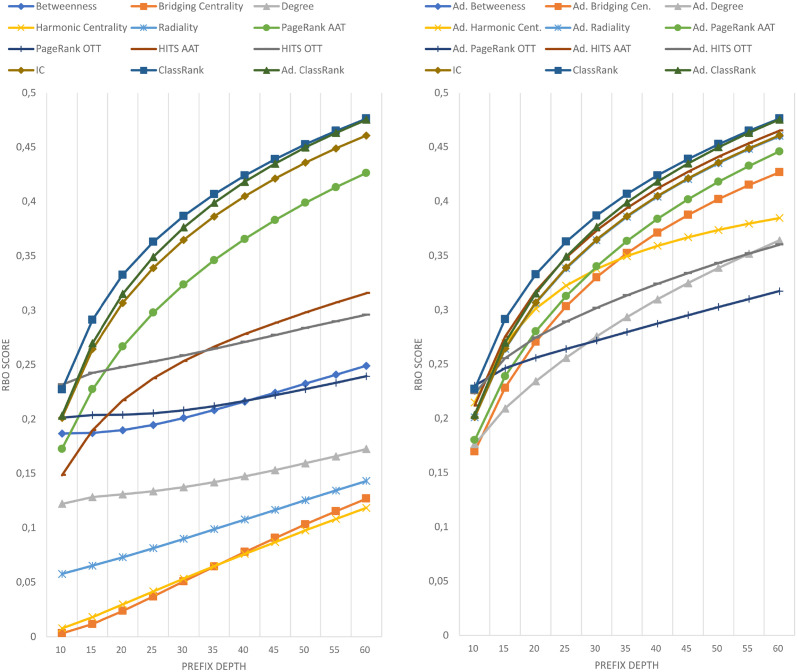
Comparison of techniques against HH log in DBpedia.

**Fig 3:**
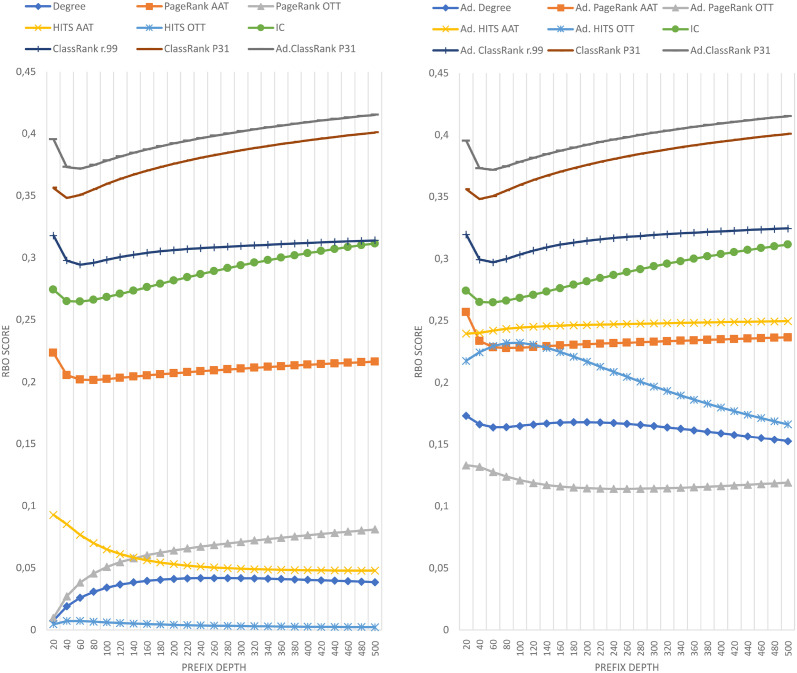
Comparison of techniques against EH log in Wikidata.

**Fig 4:**
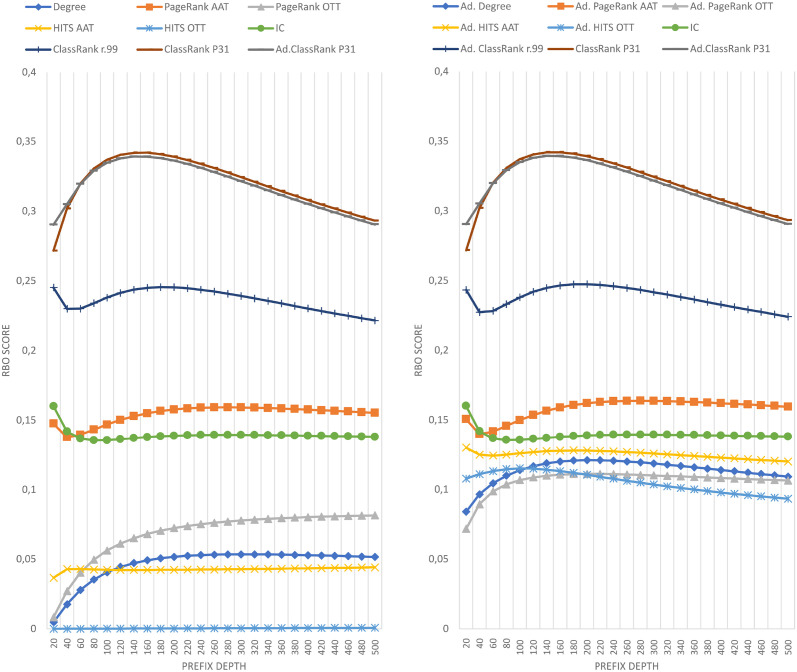
Comparison of techniques against HH log in Wikidata.

The results of comparing the metrics introduced in this paper against the importance ranking of DBpedia’s EH logs are shown in Fig 1. The results against DBpedia’s HH logs are shown in Fig 2. The results against Wikidata’s EH logs are shown in Fig 3. Finally, the results against Wikidata’s HH logs are shown in Fig 4.

Each figure contains two graphs. On the left side, we show the result of the techniques mentioned in section 3 in its raw version. On the right side, we show the adaptations of those techniques with IC scores according to the formula explained in Section 3.3. The top-performing metrics of each group are included in both sides to show the distance between any technique and the best results. IC is shown on the right graphics because the comparison between IC and any adapted technique is quite relevant. Any adapted approach outperformed by IC should be avoided, as it would be demanding extra computational cost to obtain worse results than pure IC.

The Wikidata results shown in Figs 3 and 4 include two different executions of ClassRank. We have named *ClassRank P31* the configuration in which *P*_*C*_ = {*‘P31—instance of’*}, and *ClassRank r.99* the configuration in which *P*_*C*_ includes the properties shown in Table 4.

In this paper, we focus on the comparison between the metrics’ results and the reference rankings at specific prefix depths. To allow further analysis, we have published all of our results. The complete rankings obtained during the experimentation are available on-line [39].

## 5 Discussion

### 5.1 Best performing techniques

As can be seen in Figs 1–4, there is not a metric outperforming all the rest in every case. However, ClassRank and Adapted ClassRank are the approaches performing better at mostly any source and prefix depth configuration.

According to [40], when comparing two techniques, improvements of less than 5% could be discarded and attributed to the nature of the samples chosen in the experiments; improvements between 5% and 10% are noticeable; improvements greater than 10% can be considered material.

**Fig 5:**
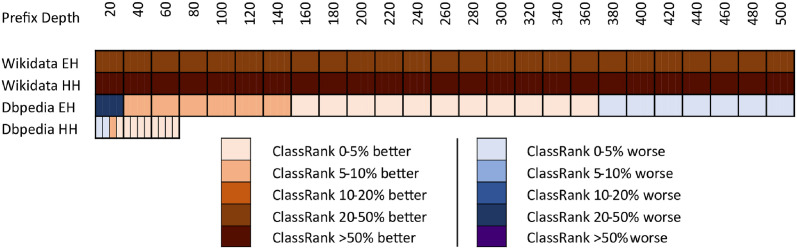
Performance comparison of ClassRank against any other non-ClassRank metric at every source and prefix depth.

**Fig 6:**
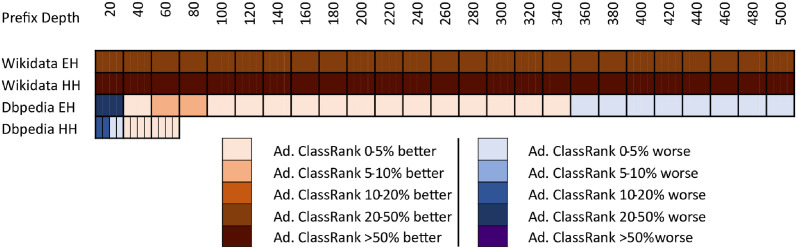
Performance comparison of Adapted ClassRank against any other non-ClassRank metric at every source and prefix depth.

In Fig 5, we show a comparison between ClassRank and the best non-ClassRank score (i.e., excluding any ClassRank configuration or Adapted ClassRank) for each source and prefix depth. The ClassRank performance curves used to make comparison in DBpedia logs are the ones labeled with *‘ClassRank’* in Figs 1 and 2. The curves used for Wikidata are the ones labeled *‘ClassRank P31’* in Figs 3 and 4. In Fig 6, Adapted ClassRank scores are compared with the best non-ClassRank score for each source and prefix depth.

Whit the criteria proposed in [40], one can see that both ClassRank and Adapted ClassRank perform materially better than any other metric for every depth explored in Wikidata logs. This is even more noticeable for HH Wikidata. As shown in Fig 4, for depth between 40 and 260, both metrics have an improvement greater than 100% over the rest of the approaches.

With DBpedia logs, even if ClassRank variants are the best-performing ones at most of the depths, this advantage is not that clear. As one can see in Fig 1, with EH entires Adapted PageRank OTT performs materially better than ClassRank. Between depth 40 and 140, ClassRank performs noticeably better than the rest of the approaches. From this depth, ClassRank performs slightly better than the second best-performing approach, which is Adapted PageRank ATT. However, the distance with this curve is always inferior to 5%. In depths beyond 380, ClassRank is slightly outperformed by Adapted Betweenness, and beyond 420 by Adapted Radiality as well.

We can observe similar circumstances with DBpedia’s HH entries. ClassRank is slightly outperformed by Adapted PageRank OTT at depth 10. Then, ClassRank performs noticeably better than the rest of the approaches at depth 15. From this point, ClassRank keeps being the best-performing approach, but with advantages lower than 5% over the second one at every depth.

The high top of DBpedia’s rankings are the only regions explored in which any technique noticeably outperforms ClassRank. That situation happens with *d* = 10 for HH entries and with *d* = 20 for EH entries. Several factors related to the topology of DBpedia Ontology cause this.

First of all, this ontology is structured as a tree in which *owl:Thing* is the root and has many direct children. Many classes do not have any children at all and, when they do, their subtrees are not deep. This structure causes *owl:Thing* to be ranked in the first position of most OTT metrics, with a great distance to the class ranked 2^nd^. On the other hand, the IC scores have lesser score differences in the top positions of the rank. Then, when the OOT metrics are adapted, IC has a more significant influence on the top spots of the adapted metrics. Also, among those classes that do have a populated subtree, we can find elements such as *dbo:Place*, *dbo:Organisation*, or *dbo:Person*. These nodes are general enough to have a subtree of classes, boosting its rank in OTT approaches, such as Degree or PageRank. At a time, these nodes are direct classes of many instances, which increases the chances of being mentioned in SPARQL logs.

These factors, combined with the relatively low number of elements in the DBpedia Ontology, make some adapted approaches to perform even better than ClassRank at the very top of DBpedia’s rankings. However, this works just for few nodes, such as the mentioned once. The deeper we go into the ranking, the better perform ClassRank and, in general, the metrics which were originally AAT.

**Fig 7:**
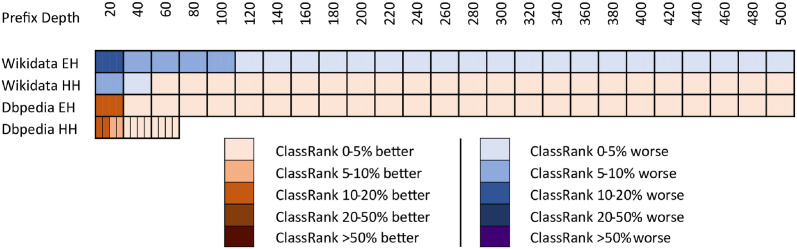
Performance comparison of ClassRank against Adapted ClassRank at every source and prefix depth.

It is hard to determine in which conditions ClassRank performs better than Adapted ClassRank and vice-versa. In Fig 7, we show the comparison between ClassRank and Adapted ClassRank. As one can see, the general case is that ClassRank outperforms its adapted version. Nevertheless, in most of the cases, the relative difference between these two approaches is lower than 5%. The only exceptions to these situations occur in the top positions of each reference ranking. Adapted ClassRank performed materially or noticeably better than ClassRank until the top 100 of Wikidata EH, and until top 20 for Wikidata HH. In opposition, ClassRank performed materially or noticeably better until top 20 of both DBpedia EH and HH. With this data, it cannot be concluded whether ClassRank outperforms Adapted ClassRank in general terms.

### 5.2 General performing of techniques in different sources

Most of the scores of any technique for any depth and source are, in general, far from 1, which would mean a perfect alignment between a reference ranking and the ranking produced by a given approach. The highest similitude between any reference ranking and any tested metric is reached by Adapted Betweenness with DBpedia’s EH entries, scoring 0.781 for a prefix depth of 500. However, except for DBpedia EH, no other reference ranking produce any measure above 0.5.

That means that there is not such a technique able to precisely capture the notion of importance observed in log’s class usage. The probable cause of this is that there is not either a perfect mathematical relation between a graph’s structure and the actual usage of its nodes in a SPARQL endpoint. Nevertheless, log usage information is not always available, so it is worth it to keep working on metrics able to find the best possible relationship between a graph’s structure and its actual usage.

With DBpedia, the lowest scores occur at the top positions of the rankings. The higher is the ranking prefix, the better is the final score. All the performance curves in Figs 1 and 2 have a linear or asymptotic-like shape with a constant improvement. That can be explained by the fact that the number of classes to rank is nearly covered by the max prefix length chosen. Then, when exploring regions of the ranking deep enough, even for pure randomness, it is easier to find classes shared by the reference list and the list under evaluation. That boosts RBO scores when considering wider prefixes.

In opposition, a prefix depth of 500 represents just 0.0002% of the Wikidata classes. Then, the chances of getting shared classes between the list compared due to randomness dramatically decrease. With Wikidata’s large number of classes to rank, there is not a general tendency with performance curves. Even within the same graphic, we observe linear, asymptotic, and parabolic-like curves. As shown in Figs 5 and 6, with a large number of classes to rank, ClassRank materially outperforms any other metric at every measured prefix depth.

### 5.3 EH vs HH results

It seems that, in general, the correlation between the graph’s structure and the class usage in SPARQL endpoints is higher with robotic agents. For a given metric, prefix depth, and source, the general case is that the HH score is lower than the EH score. Also, the general case is that the difference between these two scores is material, i.e., higher than 10%.

However, as discussed in the previous section, ClassRank and Adapted ClassRank outperform the rest of the techniques for EH and HH entries. This is, among the evaluated algorithms, ClassRank seems to be the approach that captures better the notion of importance w.r.t. class usage by both types of traffic (organic and robotic).

### 5.4 OTT, AAT and adapted metrics

With the notion of class importance adopted in this paper, the general case is that AAT techniques outperform OTT ones. There are very few exceptions to this observation. The most salient exception happens 1) with DBpedia HH entires, where HITS OTT outperforms HITS AAT until depth 35, and 2) with Wikidata, where HITS AAT scores are worse than some OTT approaches for some prefix values. This former exception takes place from depth 140 with EH entries and from depth 80 with HH entries.

Not computing the A-BOX section of the graph led to a loss of valuable knowledge about the KG’s topology. The adaptation of OTT metrics with IC scores is proposed to use, in a computationally cheap way, a salient feature of the graph, which are class-instance relations. It is remarkable that the general case for every raw metric X is that the adapted version of X outperforms raw X. That is probably because IC scores perform better than almost any other raw metric. The only exception to this is ClassRank, which is also the only technique that is not clearly outperformed by its adapted version.

It is worth mentioning that, in every case, ClassRank and Adapted ClassRank tend to describe performance curves of similar shape and, except for the top positions and the Wikidata EH log, they have score points nearly swapped. That means that, in general, the adaptation with IC scores does not have a noticeable impact over ClassRank. The probable reason for this is that several top-ranked classes appear in the tops of both IC and ClassRank. Since the top positions of the rankings have a determinant impact on RBO scores, the inclusion of IC’s well top-ranked elements boosts the results of most of the metrics. That improvement does not happen when adapting ClassRank because those key classes are already top-ranked in raw ClassRank.

### 5.5 Configuration of class-pointers

The possibility to configure the set of class-pointers *P*_*C*_ to be used during the execution of ClassRank is more linked to the concept of relevance than importance, as it is a user’s choice to let the algorithm focus on some specific aspects of the KG.

We have been able to test different *P*_*C*_ settings with Wikidata. However, due to the high amount of potential class-pointers in this source, we have not tested every possible configuration of properties, but just those described in section 4.2.2. As can be seen in Figs 3 and 4, even the best *P*_*C*_ setting performed materially worse than the straight-forward configuration *P*_*C*_ = {*‘P31—instance of’*}. Custom and more user-centered configurations of *P*_*C*_ could achieve better results.

Also, it is worth mentioning that even if *ClassRank r.99* performed worse than *ClassRank P31*, both *ClassRank r.99* and Adapted *ClassRank r.99* performed better than any other raw or adapted approach (excluding other ClassRank configurations).

### 5.6 Computational cost vs performance

In Table 6, we have included the computational cost of every technique evaluated in our experimentation.

**Table 6:** Computational cost of the techniques evaluated.

Technique	Complexity
Degree	O(n + e)
Betweenness	O(n ⋅ (n ⋅ e))
Bridging Centrality	O(n ⋅ (n ⋅ e))
Harmonic Centrality	O(n ⋅ (n + e))
Radiality	O(n ⋅ (n + e))
Instance Counting	O(n + *e*_*i*_)
PageRank	O((n + e) ⋅ $\frac{\log n}{\epsilon }$) *
HITS	O(e ⋅ k)
ClassRank	O(*n*_*c*_+ *e*_*i*_ + (n + e) ⋅ $\frac{\log n}{\epsilon }$)

n = number of nodes; e = number of edges; *e*_*i*_ = number of instantiation edges;

*n*_*c*_ = number of classes; k = maximum number of HITS iterations

* According to [41], PageRank can be computed in *ϵ* rounds, being $\frac{\log n}{\epsilon }$ the reset probability of PageRank, and having each round a complexity of O(n + e)

As one can see, Degree and IC are the cheapest techniques to execute, as they perform simple counting actions over the target nodes.

The execution of PageRank and HITS’s base operation is generally based on eigenvector computations. Even if the complexity of this is linear w.r.t to the number of nodes and edges, both approaches require several iterations until they converge and reach a result, increasing the complexity compared to Degree and IC. ClassRank has a complexity similar to PageRank because it requires PageRank scores. Once those scores are computed, the complexity of the rest of the algorithm is linear w.r.t. the number of nodes and target edges.

Betweenness, Bridging Centrality, Harmonic Centrality, and Radiality have complexities higher than quadratic w.r.t. numbers of nodes, as they all need to compute the shortest path between any pair of nodes in the target graph. This complexity makes them suitable for small or moderate-sized structures, but sometimes prohibitive in big networks.

The adapted techniques have not been included in Table 6 because they all have the same computational complexity as their raw version. That is because the adaptations purely consist of normalizing and averaging the original scores with IC scores, and IC has the lowest computational cost of all the analyzed techniques.

When choosing a metric to measure class importance, both the performance and the computational cost should be considered. Different contexts may lead to different decisions regarding whether it is preferable to prioritize performance or cost. However, some techniques could be discarded when they perform worse than their competitors w.r.t. to both decision parameters.

Even though IC is the simplest approach, it outperforms any other raw technique except ClassRank and, as one can see in Fig 1, PageRank AAT in a section of DBpedia EH ranking. Also, it is worth mentioning that, in most of the cases, the adapted version of those algorithms does not outperform IC scores. Again, the only exception to this is Adapted ClassRank and, as it is shown in Figs 1 and 2, PageRank AAT and HITS AAT is some sections of DBpedia logs.

Since the complexity of HITS, PageRank, and ClassRank is similar, but ClassRank seems to outperform HITS and PageRank, we will discuss the differences between ClassRank and IC. In Table 7, we show the top 20 elements in DBpedia according to ClassRank and IC.

**Table 7:** Top 20 of ClassRank and Instance Counting.

Rank	ClassRank	Instance Counting
**1**	dbo:Person	dbo:Person
**2**	skos:Concept	dbo:CareerStation
**3**	dbo:CareerStation	dbo:SportsTeamMember
**4**	dbo:Settlement	dbo:Settlement
**5**	dbo:SoccerClub	dbo:PersonFunction
**6**	dbo:SoccerPlayer	dbo:Village
**7**	dbo:SportsTeamMember	dbo:TimePeriod
**8**	dbo:Country	dbo:Album
**9**	dbo:City	dbo:Insect
**10**	dbo:PersonFunction	dbo:SoccerPlayer
**11**	dbo:Village	dbo:Film
**12**	dbo:AdministrativeRegion	skos:Concept
**13**	dbo:TimePeriod	dbo:OfficeHolder
**14**	dbo:Insect	dbo:Company
**15**	dbo:Album	dbo:Plant
**16**	dbo:OfficeHolder	dbo:MusicalArtist
**17**	dbo:Film	dbo:Single
**18**	dbo:Company	dbo:Building
**19**	dbo:MusicGenre	dbo:Town
**20**	dbo:MusicalArtist	dbo:Athlete

As one can see, the rankings contain elements related to very similar domains (mainly arts, sports, geopolitical divisions, and people). Fourteen classes appear at the top-20 of both lists, and some of them even have an identical rank. That makes sense since many incoming links coming from many instances ensure high importance with IC, but, frequently, they also cause high importance with ClassRank due to accumulated PageRank scores of that many instances.

A couple of missing elements in the top-20 of IC raises a clue about the kind of classes in which these two techniques heavily disagree: *dbo:SoccerClub* and *dbo:Country*. With ClassRank, *dbo:SoccerClub* ranks 5^th^ and *dbo:SoccerPlayer* ranks 6^th^. The different soccer players are connected to their club, so some of the importance accumulated by all soccer players goes to their respective teams. With this, even if there are much fewer instances of clubs (21,955) than players (117,619), these two classes can achieve a similar final score of importance. By contrast, *dbo:SoccerClub* descends to position 34^th^ with IC.

The case of *dbo:Country* is more revealing. Instances of countries are frequently key elements to link different topics in cross-domain KGs such as DBpedia. Many kinds of individuals can be linked to their country, such as smaller administrative divisions, people, geographical entities, or events. With ClassRank, that accumulated importance is good enough to rank 8^th^. *dbo:Country* is one of the top seeds according to the reference rankings as well (9^th^ with HH and 21^st^ with EH). By contrast, *dbo:Country* descends to 145^th^ with IC.

Similar examples are *dbo:MusicalGenre* (19^th^ with ClassRank vs 223^rd^ with IC), *dbo:Legislature* (42^nd^ vs 192^nd^), and *dbo:Language* (24^th^ vs 85^th^). In general, we can say that IC and ClassRank produce rankings that tend to be quite similar. However, ClassRank can capture the importance of classes that do not have too many instances when those instances are really important elements of the KG.

## 6 Related work

RDF summarization can be performed using a wide range of different techniques based in different dimensions of the target graph. Even if most of the current techniques rely at some point on concepts of node importance or relevance, some techniques, such as pattern-minning methods [42] or quotient summaries [43, 44] may not use importance metrics.

However, along this section, we will focus in those publications that explore node importance as a main goal or as a previous step to achieve another goal (commonly graph summarization).

### 6.1 Importance or relevance of entities or classes in KGs

Several authors have already used centrality metrics to determine entity importance or relevance in KGs.

In [16], a study of different techniques to detect class importance is performed. They check the performance of Degree, Betweenness, Bridging Centrality, Harmonic Centrality, Radiality and Ego Centrality against a gold standard built using DBpedia logs in the same manner that we do in this paper. Also, they propose the adaptation of the aforementioned metrics to make use of instance information which has been evaluated in our document. The study is a preliminary stage in order to support graph summarization processes. There are two main differences between this study and ours. First, the authors do nos experiment with spectral measures such as PageRank. Second, they use Spearman correlation coefficient to determine the similarity between the rankings, so the top and the tail of each ranking have the same weight on the final results.

Authors in [12] perform another general study of class importance as a previous step for ontology summarization. Again, all the techniques used are applied over the ontologies, not using A-BOX knowledge at any point.

In [13], another case of measuring class importance for ontology summarization is presented. They propose two methods, one inspired in Degree, which is defined in [45], and another one inspired in Closeness. However, those methods require some user input, such as relevant domain-specific relations in ontologies or weights for certain elements.

RDFDigest+ is a tool to perform RDF/S Knowledge Base exploration using summaries [14]. It allows the user to choose and combine many different centrality algorithms to identify the most important nodes. It also uses information related to the instances of each schema element. In [46], the same authors expose *zoom* and *extend* operations for RDFDigest+, which enable the user to get ontology summaries with different detail level in an efficient way. They perform a stage of class importance detection in which HITS, PageRank, and Betweenness are used. They also apply the adaptation of these approaches with IC scores proposed in [16].

In [47], the authors perform a study of several cross-domain KGs quality, including DBpedia and four more sources. One of the features studied is class coverage for different knowledge domains. They manually classify each class to belong to the different domains. Then, they measure the importance that each class provides to each domain by counting their instances.

In [48], PageRank is applied over a graph of Wikipedia linked entries. Each entry is represented by its DBpedia URI, so they produce a ranking of DBpedia entities based on the Wikipedia link graph. In combination with other methods, the results are being used for entity summarization [49, 50]. In order to merge the information of different Wikipedia chapters, the authors compute a ranking of Wikipedia entities using their Wikidata URI, which is unique for all the languages. In these works, PageRank is used as a base metric to rank entities in a KG. However, they use a voting method w.r.t. different Wikipedia chapters. Thus, this technique cannot be applied over KGs whose elements are not linked (directly or indirectly) to Wikipedia pages.

In [51], an approach to rank classes in DBpedia is presented. As well as ClassRank, this work is also based on aggregation of PageRank scores. Nevertheless, these scores are not obtained from DBpedia’s structure, but each entity receives the PageRank score of its associated page in Wikipedia. Then, this technique cannot be applied over KGs whose entities are not linked to Wikipedia. Also, the authors combine the aggregation of PageRank scores with some other parameters such as Instance Counting.

In [52], PageRank is applied over the DBpedia link structure to mine significant concepts. Given an element in DBpedia, the authors track its most related concepts by exploring its neighborhood in the graph, and they rank those results according to inverse PageRank. They consider that the most related elements are the ones with a lower PageRank score. The authors argue that elements with low PageRank are not so well connected because they are too specific of a given topic. Hence, those URIs in the neighborhood of a concept *c* with low PageRank may have higher chances of being semantically closer to *c* than those with high PageRank, which may be too transversal.

An approach to explain and use entity relatedness in KGs is presented in [53]. The authors formalize the concept *relatedness explanation* between two entities as a subgraph containing paths that link those two entities. Once they obtain an explanation, they are able to detect pairs of entities in the KG sharing a similar notion of relatedness. This proposal focuses on the detection and ranking of important paths between nodes. The authors compute the importance of each path using mainly properties (predicates) instead of entities. However, there is also a stage of ranking entity importance PageRank-based.

In [54], an approach to identify key concepts in ontologies is presented. This work uses a notion of importance based on experts agreement. The algorithm presented combines purely topological features of the ontology with cognitive concepts such as natural categories [55] or popularity according to results in web search systems. The approach tries to detect the elements that best summarize the semantics of the target ontology. The experiments show a high correlation between the approach’s results and the experts’ choices. This work differ from ours in their human-based notion of importance and the type of ontologies evaluated, which are small to moderate-sized (the biggest ontology used in the experiments contains 247 elements) and domain-specific.

Freebase associates a score with ranking purposes to each one of its stored entities. However, this score is not computed with PageRank, but using a simpler formula based on link counts of an entity in Freebase KG and its associated page in Wikipedia [56].

Wikidata Project maintains some special pages offering some metrics of the graph that are frequently updated [57]. Among these results, link counts of the most used elements can be found, but there are no reports about class importance or PageRank-like scores of any element.

### 6.2 Alternative centrality measures based in PageRank

The idea of using personalized versions of PageRank to adapt the algorithm to different contexts was early suggested in [15], where the original authors of PageRank suggest an adaptation called Personalized PageRank (PPR). In PPR, there is a set of restarting nodes which are the only ones that the random walker can jump to when it gets bored of following links. PPR is still widely used and several works propose ways to optimize its execution [58].

Since that proposal, many PageRank adaptations have been published. Probably the closest adaptations to our domain are those that compute aggregations of PageRank scores. A representative example of this strategy is BlockRank [59], which divides the target graph into several disjoint blocks of smaller units. An illustrative use of BlockRank-like strategies is HostRank [60], which was thought to be applied over web structures. Nevertheless, most BlockRank-like approaches are not compatible with our domain, since in RDF graphs the very same entity may be an instance of several different classes. Hence, hypothetical blocks formed by instances of the same class would not be disjoint.

Most of the PageRank adaptations have been thought to measure the relevance of an element in a KG w.r.t. a query [61]. These approaches are focused on information retrieval tasks and tend to rank entities using notions of semantic relatedness between query and resource. Some of them measure importance and are used in combination with other notions to produce some result [1, 62]. Some others measure relevance, including strategies such as text similarity or exploration of topic sub-graphs in the algorithm itself [63–67].

OntologyRank [1] is designed to rank Semantic Web Documents (SWD), such as ontologies or RDF files, which are linked to each other through their internal elements. The algorithm uses the semantics of the properties to divide them into four different categories. Then, it computes a version of PageRank where each link can be weighted w.r.t. each category. Although it follows a strategy of aggregation of PageRank-like scores, OntologyRank is designed to rank different SWDs instead of elements within a single SWD.

PopRank [62] is an adaptation of PageRank designed to be applied over a network of objects. It combines two factors to obtain the popularity of an object: a weighted PageRank in which every property has its own weight, and the PageRank of the database/web page which contains the object (Web Popularity). PopRank is thought to assign a score to every entity in the graph, i.e., there is no aggregation or grouping of individuals in some class or cluster, so the algorithms have different domains of application. Also, PopRank has a stage in which some training data should be provided by experts, which may be too costly in graphs with many properties such as DBpedia or Wikidata.

ReConRank [63] is a PageRank adaptation designed to be applied over RDF domains that combines the approaches of ResourceRank and ContextRank. ReConRank is closely related to search and retrieval domains. The ranking of entities is not applied over the whole target graph but over a sub-graph composed of certain elements that are related enough to some keywords. The scores produced are a measure of relevance w.r.t. a query instead of importance.

RareRank [64] makes use of transition scores between entities, as well as PageRank does. However, it proposes a Rational Research model to define transitions between elements aiming to simulate a human strategy of jumping from one document to another. RareRank is thought to be applied in semantic search of research documents. It relies on meta-data associated with scientific papers modeled in an ontological way, as well as topic relatedness computed with Latent Dirichlet Allocation [68]. As well as ReConRank, RareRank produces scores of relevance instead of importance. Also, the model of Rational Research should be adapted to apply it in domains different from scientific documents.

DBpediaRanker [65] describes an algorithm to rank DBpedia entities w.r.t. a query. In this case, the authors do not follow a PageRank-like approach, but they consider several different notions of similarity. This includes textual similarity, proximity to a certain set of seed nodes, or results supported by external resources, such as search engines or tagging systems. Thus, although the main goal is also the ranking of RDF resources, this approach has a specific domain of application and cannot be used to measure class importance.

TripleRank [66] is a HITS-based algorithm to rank entities w.r.t. a subject and a facet (predicate) in RDF environments. TripleRank gives a notion of relevance w.r.t. some other graph elements instead of importance per se.

DWRank [67] ranks ontology concepts in search and retrieval environments. It combines three types of notions to rank a given element: text similarity with a query, hub score within its own ontology using a reversed PageRank function, and authority of its ontology w.r.t. the rest of ontologies. The goal of the algorithm is to rank ontology members, and it works purely with T-Box elements, i.e., it does not use any instance information to produce its results.

## 7 Conclusions and future work

In this paper, we have evaluated different techniques to measure class importance in KGs based on the KG’s structure. To compare the approaches, we have performed experiments using a notion of importance based on class usage in SPARQL logs. We elaborated class rankings sorting classes w.r.t. their number of mentions in the logs and measured the similarity of those rankings with the ones produced by the evaluated techniques. This similarity has been measured using Ranking Biased Overlap.

The experiments raise several conclusions. Approaches considering just T-BOX statements are, in general, outperformed by techniques that compute A-BOX knowledge as well. ClassRank, a novel proposal, outperforms the rest of the evaluated approaches in terms of similarity with the reference rankings. Instance Counting, which is the technique requiring less computational time among all the studies approaches, outperforms every other studied metric except ClassRank and, in some cases, PageRank.

Also, it has been observed that a simple adaptation of any technique averaging its results with IC scores improves the performance of the original technique. The only exception to this is the adaptation of ClassRank scores, where it is unclear whether the adaptation with IC improves the base ClassRank scores.

A qualitative comparison of ClassRank and IC shows that they produce rankings with a high proportion of shared elements. However, Instance Counting is not able to catch the importance of really well-connected elements that do not have too many instances, such as the class *dbo:Country* in DBpedia ontology.

The evaluated approaches have been compared against rankings of class usage with log entries generated by organic agents and robotic agents. It has been shown that, in general, all the evaluated techniques align better with the machine-related log entries. Nevertheless, in our experiments, ClassRank outperforms the rest of the proposals for both organic and robotic entries.

The results obtained by ClassRank in ou experiments are promising, but more test in different types of sources are needed to contrast the conclusions reached in this paper. We consider several lines of future work:

- Extend this evaluation to more Linked Data sources, both general-purpose or domain-specific.
- Evaluate the performance of ClassRank using aggregated scores different from PageRank.
- Evaluate the performance of ClassRank as a relevance metric. We consider several options: combined use of ClassRank with blocking techniques to rank a subgraph of elements; experimentation with different sets of class-pointers related to a given topic or query; usage of weights for the base PageRank scores and each class-pointer.
